# Portal Vein Thrombosis in a 21-Year-Old Man with Membranoproliferative Glomerulonephritis and Nephrotic Syndrome

**DOI:** 10.1155/2019/3409832

**Published:** 2019-05-28

**Authors:** Ilya Seleznev, Dinara Jumadilova, Assiya Naushabayeva, Kairat Kabulbayev, Gulaiym Karashasheva, Francesca Cainelli

**Affiliations:** ^1^Department of Medicine, Nazarbayev University School of Medicine, Nur-Sultan, Kazakhstan; ^2^Research Institute of Cardiology and Medicine, Almaty, Kazakhstan; ^3^National Scientific Center for Oncology and Transplantation, Nur-Sultan, Kazakhstan

## Abstract

Membranoproliferative glomerulonephritis, one of the main causes of nephrotic syndrome, is associated with a state of hypercoagulability that leads to increased risk of thrombotic events. Portosystemic collaterals may reopen due to reversal of the flow within the existing veins and be a presenting feature of thrombosis. We describe a patient who presented with large portosystemic collaterals and signs of portal hypertension and was subsequently found to be affected by membranous proliferative glomerulonephritis. Proteinuria and microscopic haematuria in a patient with signs of portal hypertension and no pre-existing liver disease should raise the suspicion of an underlying kidney disease.

## 1. Introduction

Membranoproliferative glomerulonephritis is one of the main causes of nephrotic syndrome in adults and can cause renal failure [1]. Renal vein and deep vein thrombosis may be associated with the nephrotic syndrome [2–5], and the risk appears to depend on the duration and severity of the syndrome and to correlate with very low albumin levels. Portal vein thrombosis has been rarely reported [6–8].

## 2. Case Presentation

A 21-year-old Kazakh man was referred to the National Research Center for Oncology and Transplantation in Astana, Kazakhstan, in October 2017 because of portosystemic shunts secondary to portal vein thrombosis. The past medical history did not reveal abdominal trauma, previous surgery, omphalitis, gastrointestinal bleeding, or hepatitis. The patient had been healthy until March 2017, when he developed lumbar pain, malaise, peripheral oedema, and nausea and began to lose weight. In May 2017, he felt right upper quadrant discomfort and developed abdominal distension; an abdominal ultrasound revealed splenomegaly and ascites. Liver cirrhosis was suspected. Alanine aminotransferase and aspartate aminotransferase levels were within the normal range; HBsAg, anti-HCV, and tumour markers (CEA, Ca19-9, and alpha- fetoprotein) were negative, total protein and albumin levels were reduced (49.2 g/L and 22.6 g/L, respectively), and total cholesterol increased (6.6 mmol/L; normal 3.1-5.2). Urea, creatinine, and electrolyte levels were normal. An EGDS showed superficial distal gastritis; oesophageal or gastric varices were not detected.

In June 2017, an abdominal CT scan confirmed the presence of splenomegaly and mild ascites and detected voluminous paraoesophageal varices, left splenorenal shunts, and hepatomegaly. A Doppler ultrasound of the liver showed no flow in the portal vein and its branches and enlarged splenic vein (diameter 1.7 cm) with laminar blood flow. The patient received a provisional diagnosis of portal vein thrombosis and was started on IV heparin, which was stopped four days later because of worsening thrombocytopenia. Further serologic tests revealed subclinical hypothyroidism and hypergammaglobulinemia (25.8%); ANA and AMA were negative. A urine test showed proteinuria (1.3 to 2.0 g/L) and microscopic haematuria (18-22 RBC/high power field). The patient was started on spironolactone 100 mg/day and torasemide 5 mg/day.

In October 2017, he was admitted to the National Research Center for Oncology and Transplantation and underwent mesentericoportography, which showed hepatic arterial buffer response, an indirect sign of reduced or absent blood flow in the portal vein. An abdominal ultrasound did not visualise the portal vein and showed an enlarged spleen. Lab tests revealed mild leukopenia (3.6 WBC/*μ*L), normocytic normochromic anaemia (Hb 9.8 g/dL), thrombocytopenia (80,000 platelets/*μ*L), proteinuria (2.1 g/L), and microscopic haematuria. Further tests revealed that the patient was heterozygous for both factor V Leiden (FV) and methylenetetrahydrofolate reductase (MTHFR) genes; methionine synthase (MTR) A2756G mutation was not detected. IgM and IgG anticardiolipin antibodies and antiphospholipid antibodies were negative, and homocysteine levels were normal.

**Figure 1:**
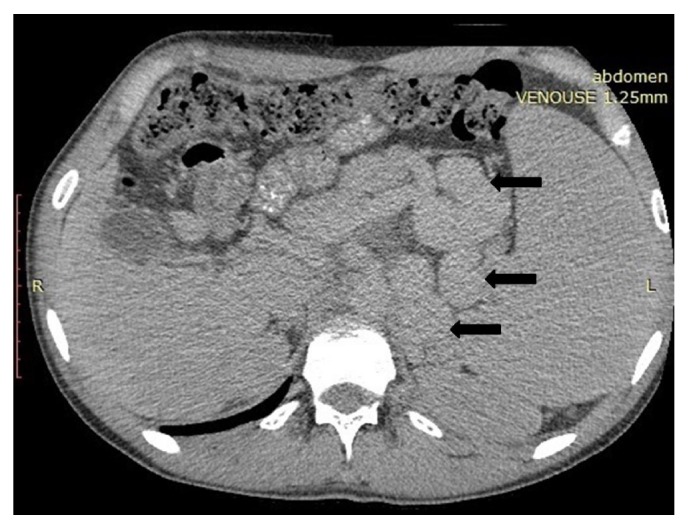
Axial postcontrast CT shows voluminous splenorenal collaterals (arrows).

A revision of the images of the CT scan performed in June 2017 revealed voluminous collaterals between the left renal and the splenic veins, paraoesophageal varices, dilatation of the superior mesenteric vein with lack of visualization of the intrahepatic portal branches, dilatation of the left renal vein with a possible filling defect, and enlarged left kidney (Figure 1).

**Figure 2:**
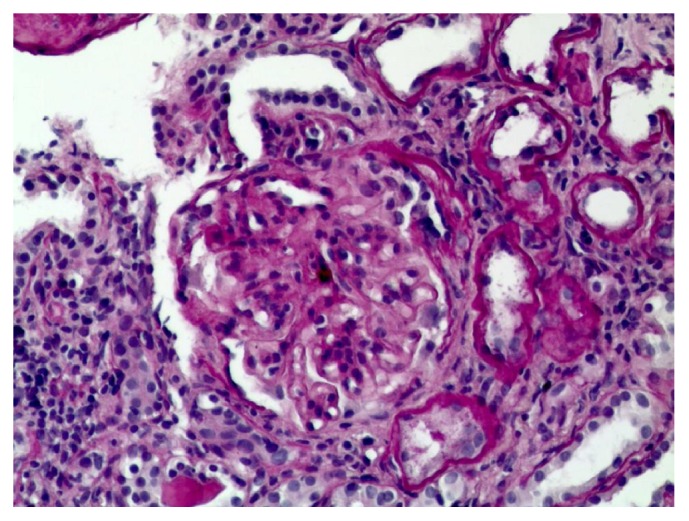
PAS x 300 Glomerulus with lobular presentation, widened mesangium, and thickened capillary walls.

**Figure 3:**
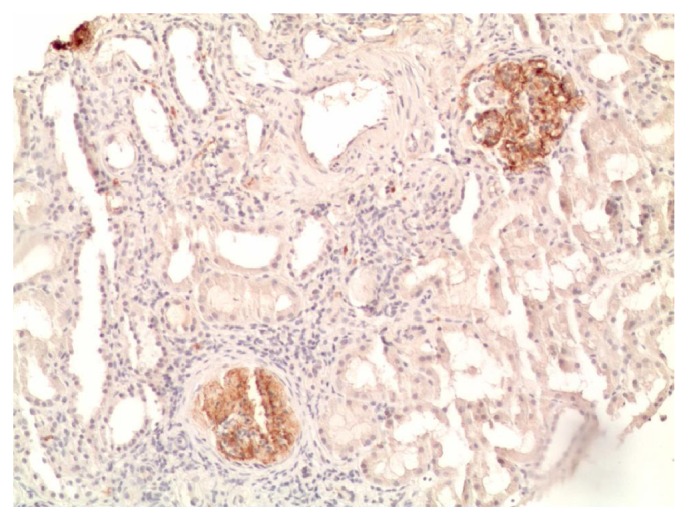
C4d x150.

In May 2018, the patient was admitted to another hospital where an echocardiography showed global enlargement of the cardiac chambers and hypertrophy of the posterior wall of the left ventricle (1.3 cm) with normal ejection fraction, II degree mitral valve regurgitation, I degree aortic regurgitation, and no signs of pulmonary hypertension. Arterial blood pressure monitoring revealed Max BP – 173/86 mm Hg, Average BP - 145/73 mm Hg, max BP (night) 141/70 mm Hg, and average BP (night) – 122/57 mm Hg. Indices of average BP were elevated during night and day, being 92% and 58%, respectively. A kidney biopsy showed moderate to severe mesangial hyper cellularity, dilation of mesangial matrix with focal lymphocytic stasis in the capillary space, basal membrane thickening of capillary loops with well-defined fuchsinophilic granules and focal total glomerulosclerosis (16%), grade II epithelial renal tubular dysplasia, diffused moderate fibrosis of the interstitium, tubular atrophy, intimal fibrosis, and hypertrophy of the arterial muscle layer of middle calibre with narrowing of one artery to 1/3 (Figures 2 and 3). A diagnosis of membranoproliferative glomerulonephritis was made and the patient was started on methylprednisolone (64 mg/day, tapering down to 16 mg/day maintenance dose) and perindopril 10 mg/day. Unfortunately the response to treatment was not optimal, leakage of ascitic fluid from the umbilicus developed, and further deterioration of the renal function led to haemodialysis. No episodes of bleeding or clinically symptomatic thrombosis occurred after the diagnosis.

## 3. Discussion

The portal vein thrombosis in our patient, who was not cirrhotic, was most likely due to the nephrotic state, a condition where depletion of intravascular volume, high fibrinogen levels and deposition as fibrin, increased platelet aggregation, urinary loss of anticoagulant factors (free Protein S, antithrombin III), and loss of fluid from the intravascular compartment with ensuing haemoconcentration contribute to a heightened thrombosis risk [9]. The few reported cases of portal vein thrombosis in nephrotic patients have indeed all occurred in males [6–8].

In our case the diagnosis of membranoproliferative glomerulonephritis was delayed as the patient presented with established signs of portal hypertension, and the presence of proteinuria and microhaematuria was not given due attention. The factor V Leiden heterozygous state might have contributed to the development of thrombosis in our patient but contrasting data have been reported in the literature with the risk being increased [10, 11] or not [12] in nephrotic patients heterozygous for FV mutation.

## 4. Conclusion

The possibility of a nephrotic syndrome should be considered when assessing a patient with unexplained portal vein thrombosis/portosystemic shunts.
